# Transcriptional Landscape of CUT-Class Homeobox Genes in Blastic Plasmacytoid Dendritic Cell Neoplasm

**DOI:** 10.3390/ijms25052764

**Published:** 2024-02-27

**Authors:** Stefan Nagel, Ulfert Rand, Claudia Pommerenke, Corinna Meyer

**Affiliations:** Department of Human and Animal Cell Lines, Leibniz-Institute DSMZ, 38124 Braunschweig, Germany

**Keywords:** ALCL, AML, CUT-code, homeodomain, HOXA9, NKL-code, pyroptosis, ZHX2

## Abstract

Homeobox genes encode developmental transcription factors regulating tissue-specific differentiation processes and drive cancerogenesis when deregulated. Dendritic cells (DCs) are myeloid immune cells occurring as two types, either conventional or plasmacytoid DCs. Recently, we showed that the expression of NKL-subclass homeobox gene VENTX is restricted to conventional DCs, regulating developmental genes. Here, we identified and investigated homeobox genes specifically expressed in plasmacytoid DCs (pDCs) and derived blastic plasmacytoid dendritic cell neoplasm (BPDCN). We analyzed gene expression data, performed RQ-PCR, protein analyses by Western blot and immuno-cytology, siRNA-mediated knockdown assays and subsequent RNA-sequencing and live-cell imaging. Screening of public gene expression data revealed restricted activity of the CUT-class homeobox gene CUX2 in pDCs. An extended analysis of this homeobox gene class in myelopoiesis showed that additional CUX2 activity was restricted to myeloid progenitors, while BPDCN patients aberrantly expressed ONECUT2, which remained silent in the complete myeloid compartment. ONECUT2 expressing BPDCN cell line CAL-1 served as a model to investigate its regulation and oncogenic activity. The ONECUT2 locus at 18q21 was duplicated and activated by IRF4, AUTS2 and TNF-signaling and repressed by BMP4-, TGFb- and IL13-signalling. Functional analyses of ONECUT2 revealed the inhibition of pDC differentiation and of CDKN1C and CASP1 expression, while SMAD3 and EPAS1 were activated. EPAS1 in turn enhanced survival under hypoxic conditions which thus may support dendritic tumor cells residing in hypoxic skin lesions. Collectively, we revealed physiological and aberrant activities of CUT-class homeobox genes in myelopoiesis including pDCs and in BPDCN, respectively. Our data may aid in the diagnosis of BPDCN patients and reveal novel therapeutic targets for this fatal malignancy.

## 1. Introduction

Transcription factors (TFs) are key players in the developmental control of hematopoiesis, constituting gene regulatory networks which, in turn, orchestrate differentiation processes [1,2,3]. Homeobox genes encode TFs sharing the so-called homeodomain which acts as a platform for both DNA and protein interactions [4]. This domain belongs to the helix-turn-helix family of TF motifs and consists of 60 amino acid residues, forming three alpha-helices. According to sequence similarities in the encoded homeodomain, homeobox genes fall into eleven classes (e.g., ANTP, CUT and TALE) and several subclasses (e.g., NKL) [5]. Homeodomain TFs play basic roles in tissue development and cell differentiation, including hematopoiesis [6,7,8]. To evaluate their role in normal and aberrant lymphoid and myeloid immune and blood cell development, we previously identified so-called “TF-codes”. These codes describe physiological activities of TALE-class or NKL-subclass homeobox genes, for example, in developing and mature hematopoietic entities, and hence are named “TALE-code” or “NKL-code” [7,9,10]. These benchmark gene signatures illuminate our understanding of regulatory processes in the hematopoietic compartment and enable rapid identification of deregulated homeobox genes in corresponding malignancies [7,9,10]. 

Dendritic cells (DCs) are myeloid immune cells which sense and capture bacteria or virus-infected cells and subsequently display specific antigens to T-cells prior to eliciting immune responses [11]. According to their function, DCs are present in most tissues and peripheral blood. Hematopoietically, they derive from either specific progenitors or monocytes. Two types of progenitor-derived DCs are distinguished, namely myeloid or conventional DCs (cDCs) and plasmacytoid DCs (pDCs). They differ in expression of specific TFs, surface markers and cytokine production [12,13,14]. 

Recently, we identified the physiological expression of the NKL-subclass homeobox gene *VENTX* exclusively in cDCs and the aberrant expression in acute myeloid leukemia (AML) [15]. Here, we report on two CUT-class homeobox genes in pDCs: *CUX2* physiologically expressed only in pDCs and *ONECUT2* aberrantly activated in blastic plasmacytoid dendritic cell neoplasm (BPDCN). CUT-class members share a specific homeodomain and one-to-three CUT domains. CUT domains comprise about 180 amino acid residues and create five alpha-helices. They also perform DNA and protein interactions, thus extending the regulatory potential of these TFs [16,17]. The CUT-class of homeobox genes is divided into three groups, CUX, ONECUT and SATB, and the human genome contains seven CUT-class genes, namely *CUX1*, *CUX2*, *ONECUT1*, *ONECUT2*, *ONECUT3*, *SATB1* and *SATB2* [4]. *ONECUT2* is normally expressed in the liver, pancreas and brain [18,19]. The aberrant expression of *ONECUT2* has been reported in prostate and lung cancer, demonstrating the oncogenic potential of this factor when deregulated [20,21,22].

BPDCN is a rare hematopoietic malignancy characterized by clonal proliferation of immature pDCs. Diagnostically, BPDCN has to be distinguished from AML [23]. The clinical manifestations of BPDCN are predominant cutaneous involvement with subsequent or simultaneous extension to the bone marrow and peripheral blood while normal pDCs are absent from the skin. The clinical course of BPDCN patients is almost invariably aggressive and fatal [24,25]. At present, there is no consensus on the optimal treatment of this disease, highlighting the urgency of identifying novel therapeutic options. Our data may improve the diagnosis and therapy of BPDCN.

## 2. Results

### 2.1. Homeobox Gene Expression in cDCs and pDCs

**Figure 1:**
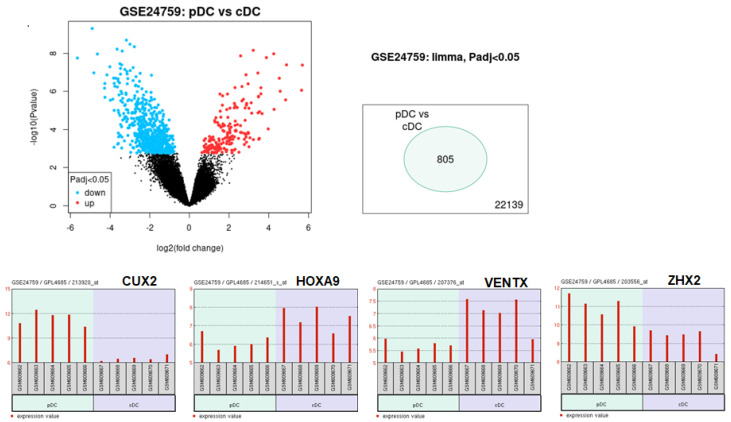
Expression of homeobox genes in DCs. Comparative gene expression profiling analysis of primary pDC and cDC samples using public dataset GSE24759 and online tool GEOR. Genes showing significant differences in their expression level (N = 805) are indicated in blue and red in the volcano plot (above). Homeobox genes *CUX2*, *HOXA9*, *VENTX* and *ZHX2* exhibited differential expression (below).

Homeobox genes underpin normal differentiation processes and drive cancerogenesis when deregulated. While investigating NKL homeobox genes in DC development, we recently reported the expression of *VENTX* exclusive to progenitor-derived cDCs [15]. To extend the search for homeodomain TFs involved in DC differentiation, we here screened for homeobox genes differentially expressed in mature DCs. To this end, we compared gene expression profiling data for cDCs and pDCs using public dataset GSE24759 and the associated online tool GEOR, thus revealing 805 genes showing statistically significant differences. These genes included four homeobox genes: *CUX2* and *ZHX2*, specifically expressed in pDCs, and *HOXA9* and *VENTX* activated in cDCs (Table S1, Figure 1). Interestingly, these homeobox genes fall into different groups: *CUX2* is a CUT-class member, *ZHX2* belongs to the ZHX-family, *HOXA9* is part of the HOXA-cluster, and *VENTX* is a member of the NKL-subclass. 

This observation prompted transcriptional analysis of all homeobox gene members in normal immune cells for each of these four groups using RNA-seq data from the Human Protein Atlas and additionally in BPDCN and AML patients using the expression profiling dataset GSE89565 (Figure S1). This revealed aberrantly expressed, closely related homeobox genes, notably the CUT-class homeobox gene *ONECUT2*, which, though silent in all mature immune cells, was aberrantly activated in BPDCN but not AML patients. Moreover, *CUX2* activity was restricted to pDCs, underlining the role of CUT-class homeobox genes in normal and malignant pDCs. 

**Figure 2:**
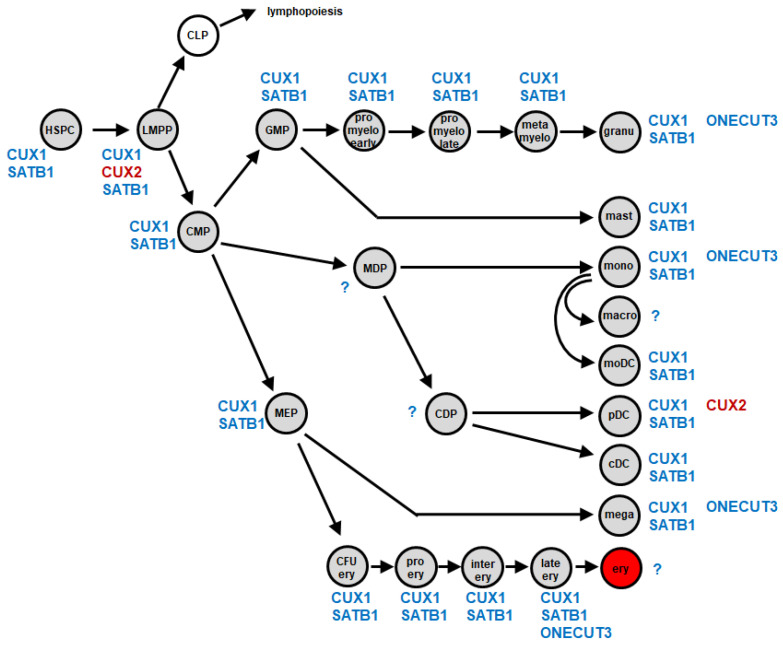
Expression of CUT-class homeobox genes in myelopoiesis (myeloid CUT-code). Schematic presentation of developing myeloid cells, including stem cells, progenitors and mature myeloid immune and blood cells, and of CUT-class homeobox genes expressed in each entity (blue, CUX2 in red). This gene signature was termed the myeloid CUT-code. Abbreviations: cDC, conventional dendritic cell; CDP, common dendritic progenitor; CFU ery, erythroid colony forming unit; CLP, common lymphoid progenitor; CMP, common myeloid progenitor; ery, erythrocyte; GMP, granulo-myeloid progenitor; granu, granulocyte; HSPC, hematopoietic stem and progenitor cell; inter ery, intermediate stage erythroblast; late ery, pyknotio-stage erythroblast; LMPP, lymphomyelo-primed progenitor; macro, macrophage; mast, mast cell; MDP, monocyte dendritic cell progenitor; mega, megakaryocyte; MEP, megakaryocytic-erythroid progenitor; metamyelo, metamyelocyte; moDC, monocyte-derived dendritic cell; mono, monocyte; pDC, plasmacytoid dendritic cell; pro ery, pro-erythroblast; pro myelo early/late, early/late promyelocyte; ?, no data available.

To focus on CUT-class homeobox genes in hematopoietic development, we performed gene expression analyses for all seven CUT-class members in myelopoiesis and identified genes active in myeloid progenitors and mature immune cells (Figure S2). We used public datasets and applied cutoffs reported in our previous studies [10]. The gene signature thus generated was termed “myeloid CUT-code” and is shown in Figure 2. This code demonstrated that *CUX1* and *SATB1* were expressed in all entities analyzed. *CUX2* was expressed in pDCs and additionally in lymphoid and myeloid primed progenitors (LMPPs), while *ONECUT1*, *ONECUT2* and *SATB2* remained silent in the complete myeloid compartment. *ONECUT3* activity was restricted to mature myeloid cells including granulocytes, monocytes and megakaryocytes. Of note, there is an ongoing discussion about the course of DC development [26]. In mice, cDCs derive from DC progenitors, while pDCs may derive from CLPs, thus representing lymphoid cells [27]. Our presented system for human myelopoiesis depicts the classical model which may require correction when this puzzle is solved [13]. Taken together, our survey of homeobox genes in DCs and myelopoiesis revealed a specific role of CUT-class homeobox genes *CUX2* and *ONECUT2* in pDCs and the derived malignancy BPDCN, respectively. In the following, we focused on these two genes in BPDCN. 

### 2.2. CUT-Class Homeobox Genes CUX2 and ONECUT2 in BPDCN Patients and Cell Lines

**Figure 3:**
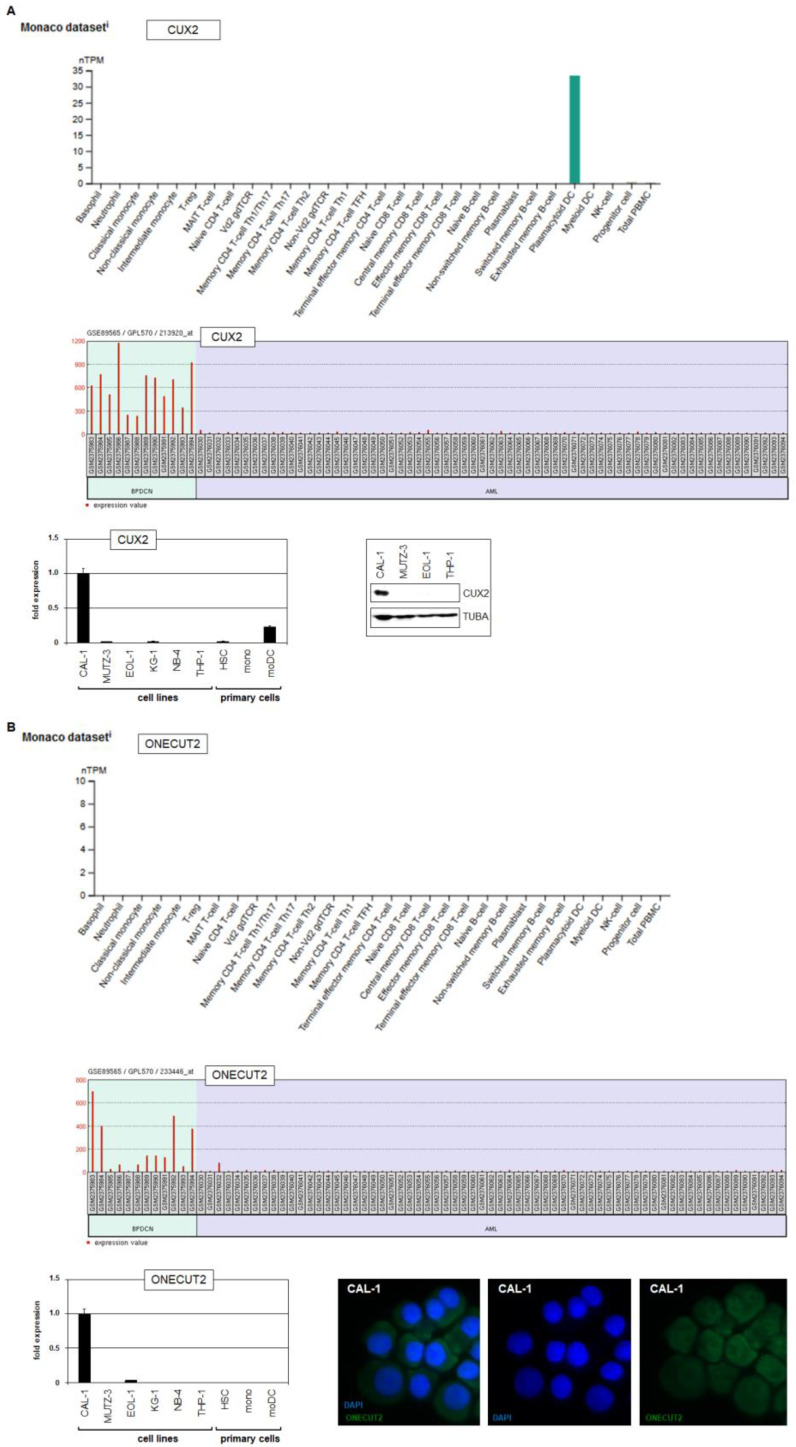
Expression of CUX2 and ONECUT2 in hematopoiesis and BPDCN. (**A**) RNA-seq gene expression data for mature immune cells from the Human Protein Atlas shows *CUX2* expression exclusively in myeloid or plasmacytoid DCs (above). Gene expression profiling data for BPDCN and AML patients from dataset GSE shows *CUX2* expression exclusively in BPDCN patients (middle). RQ-PCR and Western blot analyses show CUX2 expression in pDC cell line CAL-1 and primary DCs (below). (**B**) RNA-seq gene expression data for mature immune cells from the Human Protein Atlas discounts *ONECUT2* expression in these cells (above). Gene expression profiling data for BPDCN and AML patients from dataset GSE shows *ONECUT2* expression exclusively in BPDCN patients (middle). RQ-PCR and immuno-cytology analyses (magnification 400×) show ONECUT2 expression exclusively in pDC cell line CAL-1 (below).

The gene activities of *CUX2* and *ONECUT2* in normal immune cells and abnormal BPDCN cells are shown in Figure 3, highlighting their potential role in physiological and malignant pDCs. To find *CUX2* and *ONECUT2* positive models for further analyses and experiments, we screened 100 leukemia/lymphoma cell lines using our benchmark RNA-seq dataset LL-100 (Figure S3). The data indicated the significant expression of *CUX2* in selected B-cell lines derived from Burkitt lymphoma (RAMOS), diffuse large B-cell lymphoma (SU-DHL-4, WSU-DLCL2) and Hodgkin lymphoma (DEV). *ONECUT2* was significantly expressed in cell lines derived from anaplastic large cell lymphoma (SR-786, SUP-M2), AML (NOMO-1, OCI-AML3), Hodgkin lymphoma (HDLM-2, KM-H2, L-1236, L-428) and multiple myeloma (LP-1, U-266). These data indicate *CUX2* and *ONECUT2* as potential oncogenic drivers in subtypes of specific lymphoid and myeloid malignancies. 

Since LL-100 excludes BPDCN models, we adopted the BPDCN cell line CAL-1 established in 2005 from a 70-year-old male diagnosed with blastic NK-cell lymphoma which is now called BPDCN [28,29]. A combined analysis of public gene expression profiling datasets containing CAL-1 (GSE112209) and 20 AML control cell lines (GSE59808) indicated *CUX2* and *ONECUT2* activity in CAL-1 (Table S2). Furthermore, these data showed raised expressions of *TCL1*, *TCF4*, *CD303/CLEC4C*, *CD303/NRP1* and *NCAM1* and downregulation of *MPO*, *CD3* and *CD34* for CAL-1, corresponding to reported activities of these established BPDCN biomarkers [23,30]. In addition, we performed RQ-PCR analysis of selected cell lines, including CAL-1 (pDC/BPDCN), MUTZ-3 (cDC/AML), and EOL-1, KG-1 and THP-1 (AML), and of primary cell samples including hematopoietic stem cells, monocytes and DCs (Figure 3). Accordingly, *CUX2* was expressed in CAL-1 and primary DCs, while *ONECUT2* expression was restricted to CAL-1. Western blot and immuno-cytology analyses confirmed CUX2 and ONECUT2 expression in CAL-1 at the protein level (Figure 3). Taken together, cell line CAL-1 expressed both CUT-class homeobox genes *CUX2* and *ONECUT2* in addition to basic BPDCN biomarkers, endorsing its use as a model to investigate the potential oncogenic role of *ONECUT2* in BPDCN. 

### 2.3. Deregulation of ONECUT2 in BPDCN Cell Line CAL-1

**Figure 4:**
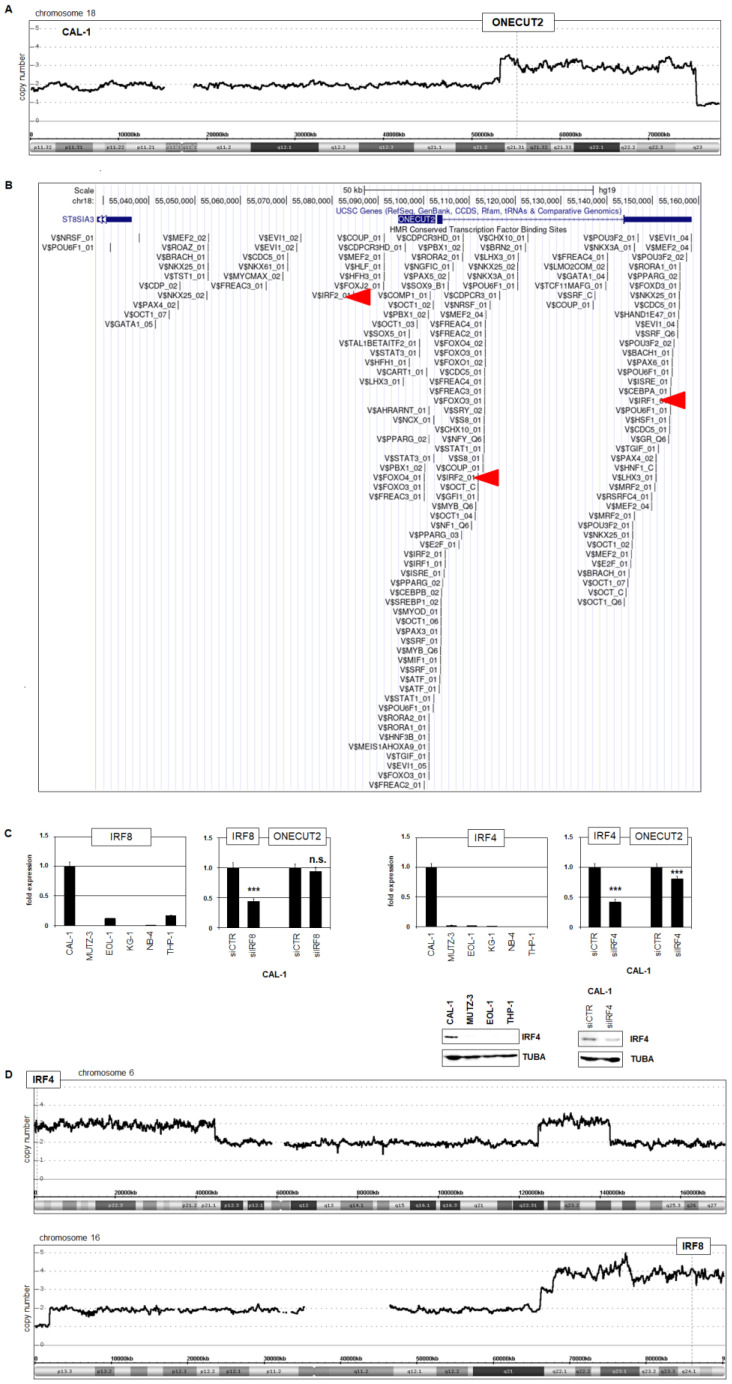
Genomic activation of *ONECUT2* in BPDCN. (**A**) Genomic profiling analysis of CAL-1 indicates duplication of 18q21-q23 targeting the *ONECUT2* locus at 18q21.31. (**B**) Analysis of TF binding sites at *ONECUT2* using the UCSC genome browser revealed potential impacts of IRF factors. Potential IRF-binding sites are indicated by red arrow heads. (**C**) RQ-PCR analysis of *IRF8* and *IRF4* in selected cell lines showed elevated activities exclusive to CAL-1. SiRNA-mediated knockdown experiments revealed an activatory role of IRF4, while IRF8 failed to regulate ONECUT2 expression. p-values are indicated by asterisks (*** *p* < 0.001, n.s. not significant). Western blot analysis confirmed IRF4 expression at the protein level in CAL-1 cells and its knockdown after siRNA treatment (below). (**D**) Genomic profiling analysis of CAL-1 indicated copy number gains for *IRF4* at 6p25 and *IRF8* at 16q24, probably underlying their overexpression.

In hematopoietic malignancies, chromosomal and genomic rearrangements frequently underlie aberrant oncogene activation [31,32]. The *ONECUT2* locus is located at chromosomal band 18q21. However, karyotypes reported for BPDCN patients and cell line CAL-1 discount recurrent rearrangements at that locus [28,33,34]. To identify genomic copy number alterations in CAL-1, we performed genomic profiling (Figure S4). These data revealed genomic duplication of the *ONECUT2* locus in CAL-1 cells (Figure 4A), plausibly supporting its aberrant expression. 

To identify TFs involved in the regulation of *ONECUT2* in CAL-1, we screened data from the UCSC genome browser showing potential TF binding sites at *ONECUT2* (Figure 4B). The results indicated the potential regulatory impact of IRF factors via three binding sites. Our comparative cell line gene expression profiling data showed elevated expression of pDC-associated *IRF8* and *IRF4* in CAL-1 (Table S2), which was confirmed by RQ-PCR analysis (Figure 4C). SiRNA-mediated knockdown experiments discounted such a role for IRF8 while demonstrating an activating impact of IRF4 for *ONECUT2* expression (Figure 4C). Moreover, our genomic profiling data showed genomic gains including *IRF4* at 6p25 and *IRF8* at 16q24 plausibly underlying their elevated expression (Figure 4D). Taken together, the aberrant expression of *ONECUT2* in BPDCN cell line CAL-1 is driven by focal genomic aberrations, respectively, targeting *ONECUT2* and *IRF4*—the latter operating as a transcriptional activator. 

To identify additional factors regulating *ONECUT2* expression in BPDCN, we analyzed the gene expression profiling data of CAL-1 in comparison to AML control cell lines in more detail (Table S2). By scrutinizing activity of the top-1000 differentially overexpressed and downregulated genes, we observed raised levels of *SMAD9*, *TNF*, *TNFRSF11A*, *CHRDL1* and *AUTS2* and reduced expression of *BMP2K*, *SMAD1* and *FGFR1*. These findings indicated potential impacts of BMP/TGFb-, FGF- and TNF-signaling and of chromatin-regulator AUTS2. 

**Figure 5:**
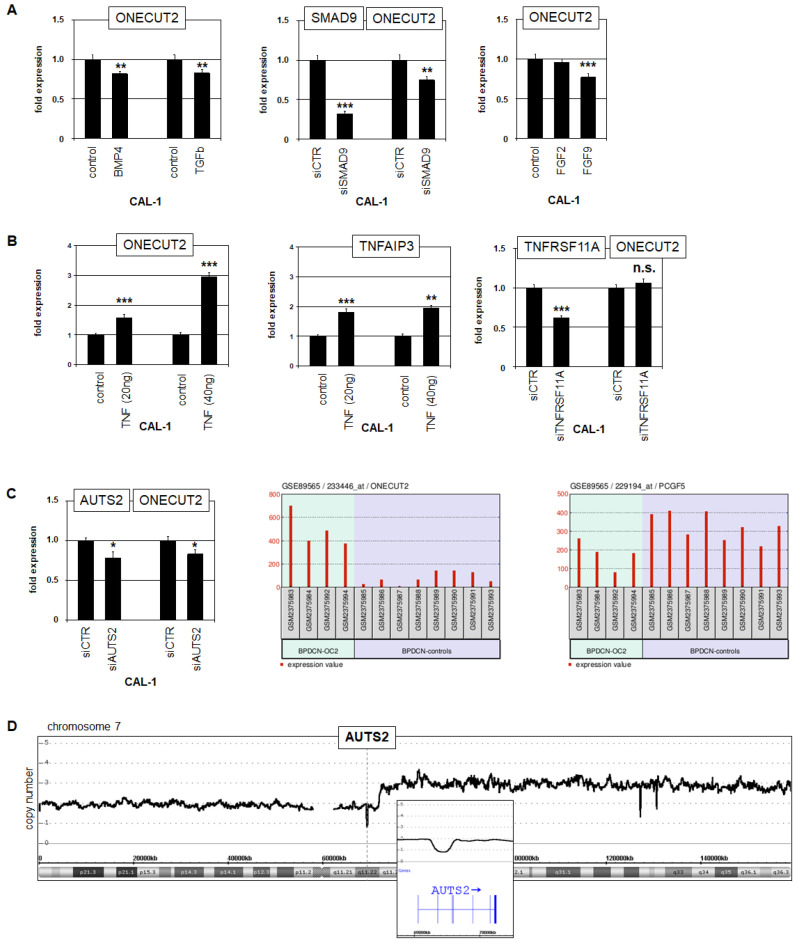
Regulation of *ONECUT2* in BPDCN by signaling pathways and chromatin factors. (**A**) RQ-PCR analyses of *ONECUT2* in CAL-1 cells after treatment with BMP4 and TGFb (left), siRNA-mediated knockdown of SMAD9 (middle) and treatment with FGF2 and FGF9 (right). (**B**) RQ-PCR analyses of *ONECUT2* and *TNFAIP3* in CAL-1 cells after treatment with TNF (left and middle) and siRNA-mediated knockdown of *TNFRSF11A* (right). (**C**) RQ-PCR analyses of *ONECUT2* in CAL-1 cells after knockdown of AUTS2 (left). Comparative gene expression profiling data of BPDCN patients expressing high and low/controls *ONECUT2* levels for the genes *ONECUT2* (middle) and *PCGF5* (right). (**D**) Genomic profiling data for chromosome 7 from BPDCN cell line CAL-1, showing a micro-deletion at the *AUTS2* locus (enlargement inserted). p-values are indicated by asterisks (* *p* < 0.05, ** *p* < 0.01, *** *p* < 0.001, n.s. not significant).

We tested the impacts of these factors by treatment of CAL-1 cells with BMP4 and TGFb which resulted in slightly reduced *ONECUT2* expression, while siRNA-mediated knockdown of the BMP-signaling inhibitor SMAD9 reduced *ONECUT2* activity (Figure 5A). FGF2-treatment showed no effect, while FGF9 inhibited *ONECUT2* expression significantly (Figure 5A). Treatment of CAL-1 with TNF activated *ONECUT2* together with the TNF-control gene *TNFAIP3*. However, siRNA-mediated knockdown of overexpressed TNFRSF11A failed to impact *ONECUT2* expression (Figure 5B). Finally, siRNA-mediated knockdown of AUTS2 inhibited expression of *ONECUT2*, indicating an activating role (Figure 5C). Interestingly, our genomic profiling data showed an intragenic deletion of *AUTS2* which may support its expression and/or activity (Figure 5D). Moreover, *ONECUT2*-positive BPDCN patients expressed reduced levels of chromatin-repressor PCGF5, which reportedly competes with chromatin-activator AUTS2 (Figure 5C, Table S3) [35]. Taken together, our results indicated that TNF-signaling activated while BMP4-, FGF9- and TGFb-signaling inhibited *ONECUT2* expression in BPDCN cell line CAL-1. Altered activities of these pathways thus may contribute to aberrant *ONECUT2* expression. Furthermore, AUTS2 and PCGF5 may (de)regulate *ONECUT2* activity via chromatin modifications in the tumor cells. 

### 2.4. Oncogenic Activities of ONECUT2 in BPDCN

**Figure 6:**
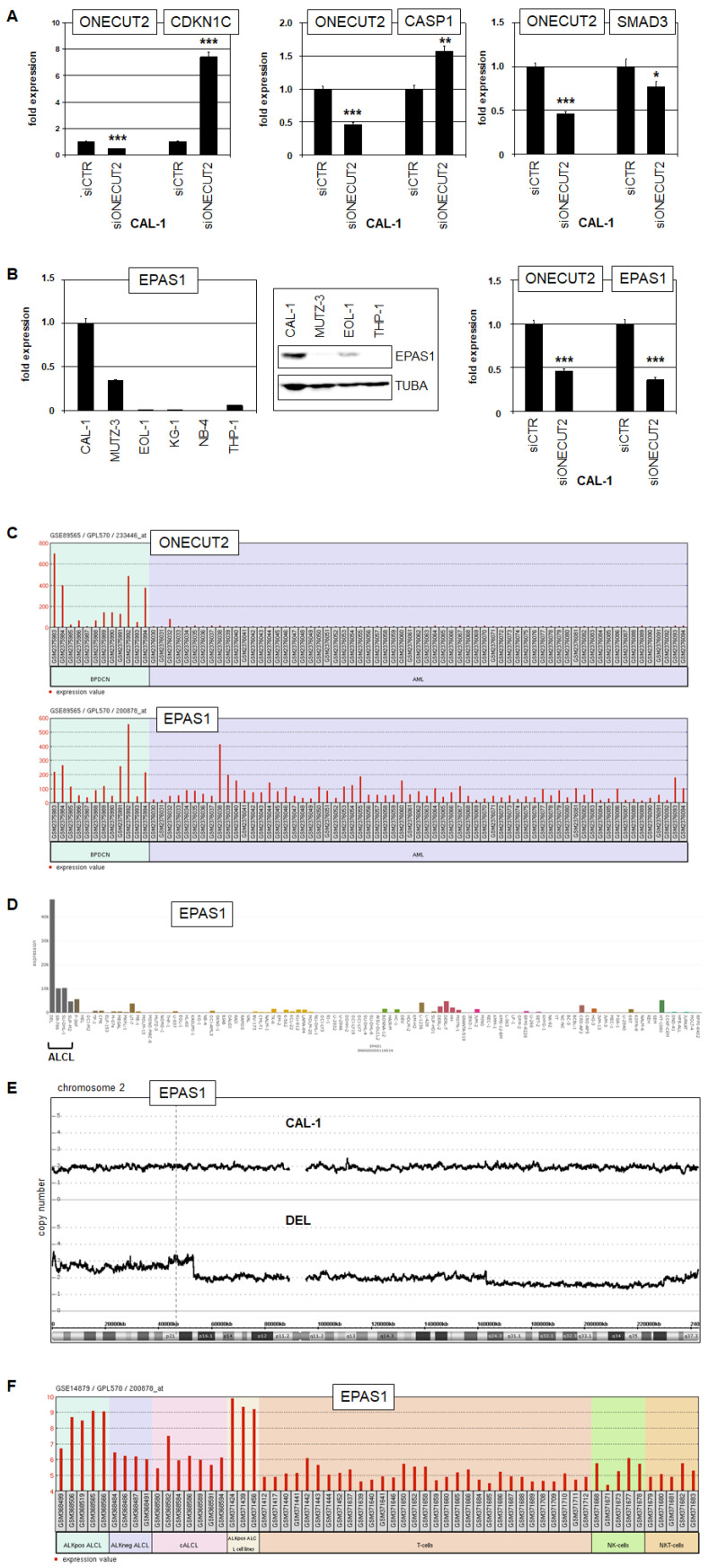
Target genes of ONECUT2 in BPDCN. (**A**) RQ-PCR analysis of *CDKN1C* (left), *CASP1* (middle) and *SMAD3* (right) in CAL-1 cells after siRNA-mediated knockdown of ONECUT2. (**B**) RQ-PCR (left) and Western blot analysis (middle) of EPAS1 in selected cell lines. RQ-PCR analysis of *EPAS1* after knockdown of ONECUT2 in CAL-1 (right). p-values are indicated by asterisks (* *p* < 0.05, ** *p* < 0.01, *** *p* < 0.001). (**C**) Gene expression profiling data for *ONECUT2* (above) and *EPAS1* (below) in BPDCN and AML patients using dataset GSE89565. (**D**) RNA-seq data for *EPAS1* in 100 leukemia/lymphoma cell lines (E-MTAB-7721), showing elevated expression levels in ALCL cell lines. (**E**) Genomic profiling data for chromosome 2 from BPDCN cell line CAL-1 and ALCL cell line DEL, showing duplication of the *EPAS1* locus in DEL. (**F**) Gene expression profiling data for *EPAS1* in ALCL patients and cell lines and T-cell controls, using dataset GSE14879. Elevated *EPAS1* expression is detectable just in ALK-positive ALCL samples.

In prostate cancer, *ONECUT2* operates as an oncogene by activating expression of *SMAD3* which regulates tumor growth under hypoxic conditions [21]. Here, siRNA-mediated knockdown experiments in CAL-1 demonstrated that ONECUT2 activated *SMAD3* in BPDCN as well (Figure 6A). Furthermore, our comparative gene expression profiling data indicated reduced expression of *CDKN1C* and *CASP1* in CAL-1 (Table S2). CDKN1C inhibits proliferation and CASP1 mediates pyroptosis—a form of cell death triggered by proinflammatory signaling, thus representing tumor suppressor genes [36,37]. Knockdown of *ONECUT2* resulted in elevated expression of *CDKN1C* and *CASP1*, demonstrating that ONECUT2 inhibits both genes (Figure 6A). 

To analyze ONECUT2 target genes in BPDCN systematically, we performed RNA sequencing of CAL-1 cells treated for ONECUT2 knockdown in triplicate. The most significantly altered genes are listed in Table 1. Here, we focused on *EPAS1* which encodes a factor orchestrating the cellular physiology under hypoxic conditions [38]. RQ-PCR and Western blot analyses showed elevated *EPAS1* levels in CAL-1 and siRNA-mediated knockdown experiments confirmed the activating impact of ONECUT2 on *EPAS1* (Figure 6B). Furthermore, expression of *EPAS1* and its cofactors *ARNT* and *ARNTL* correlated with *ONECUT2* in BPDCN patients and cell line CAL-1, respectively, supporting functional relevance of these findings (Tables S2 and S3). 

Interestingly, LL-100 RNA-seq data of leukemia/lymphoma cell lines demonstrated elevated *EPAS1* expression in ONECUT2-positive anaplastic large cell lymphoma (ALCL)-derived cell lines (Figure 6D). In contrast to BPDCN cell line CAL-1, ALCL cell line DEL showed a genomic gain at 2p21 hosting the *EPAS1* locus (Figure 6E). Thus, aberrantly enhanced *EPAS1* expression is driven by genomic rearrangement in ALCL subsets and by ONECUT2, likely affecting both BPDCN and ALCL (Figure S3). Moreover, *EPAS1* overexpression correlated with the presence of hallmark fusion gene *NPM1::ALK* in ALCL patients and cell lines (Figure 6F). Taken together, we identified specific target genes of ONECUT2 in BPDCN, including repressed *CDKN1C* and *CASP1*, and activated *SMAD3* and *EPAS1*. SMAD3 reportedly supports the growth of prostate cancer cells under hypoxic conditions [21], further supporting the notion that hypoxic adaptation may represent an important oncogenic function of ONECUT2 and its target genes *SMAD3* and *EPAS1* in BPDCN. 

**Figure 7:**
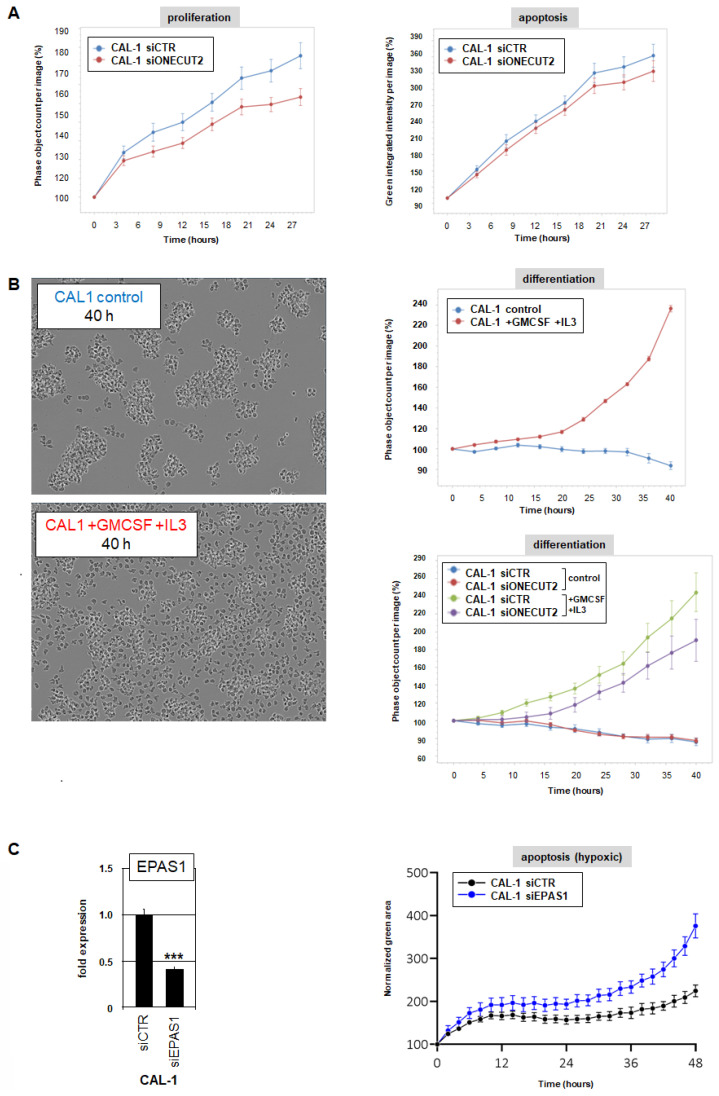
Functional analyses of ONECUT2 in BPDCN cell line CAL-1. (**A**) Analysis of proliferation (left) and apoptosis (right) in CAL-1 cells treated for siRNA-mediated knockdown of ONECUT2 by live-cell imaging. Statistical analysis of the last time point of recording using two-tailed *t*-test revealed significant differences for proliferation (*p* = 0.0433) but not for apoptosis. (**B**) Treatment of CAL-1 cells with GM-CSF and IL3 induced DC differentiation detectable by reduced aggregation of the cells as shown by live-cell images after 40 h (left, magnification 400×). Digital analysis of live-cell images allowed quantification of cell clumping over time: clumped cells were not discriminated leading to decreased cell counts. This approach demonstrated GM-CSF/IL3-induced DC differentiation of CAL-1 which was significantly reduced after ONECUT2 knockdown (two-tailed *t*-test of last time point of recording: *p* = 0.0185). (**C**) RQ-PCR analysis of *EPAS1* after siRNA-mediated knockdown of *EPAS1* (left). p-value is indicated by an asterisk (*** *p* < 0.001). Quantification of apoptotic cells by live-cell imaging analysis of CAL-1 treated for *EPAS1* knockdown and cultivated under hypoxic conditions. Statistical analysis of the area under curve revealed a significant increase of apoptotic cells after *EPAS1* knockdown (*p* = 0.0016). All live-cell imaging experiments were repeated twice, generating similar results.

Next, we analyzed the function of ONECUT2 in BPDCN cell line CAL-1 by live-cell imaging. SiRNA-mediated knockdown of ONECUT2 reduced the proliferation but spared survival (Figure 7A). Thus, ONECUT2 supports proliferation, correlating with repression of cell cycle inhibitor CDKN1C. 

Treatment of pDC-derived cell line CAL-1 with GM-CSF (alias CSF2) and IL3 overcomes its pathological differentiation arrest, continuing the process of DC maturation [28]. Undifferentiated CAL-1 cells aggregate in culture while treatment with GM-CSF and IL3 suppressed this behavior, thus serving as a surrogate to quantify DC differentiation (Figure 7B). The used software tool analyzing live-cell imaging data is unable to identify single cells located in clusters, resulting in reduced cell counts. GM-CSF/IL3 stimulation dispersed the cell aggregates, resulting in increased cell counts. Accordingly, siRNA-mediated knockdown of ONECUT2 enhanced cell aggregation inhibited by GM-CSF/IL3 stimulation (Figure 7B), indicating that ONECUT2 repressed DC differentiation processes. 

Finally, to investigate the potential role of the ONECUT2 target gene *EPAS1* in cell survival under hypoxic conditions, we reduced the normal oxygen level (20%) to 5%. *EPAS1* expression levels were reduced in CAL-1 cells by siRNA-mediated knockdown. Subsequent quantification of apoptotic cells by live-cell imaging demonstrated that EPAS1 supported survival of BPDCN cells growing with reduced oxygen (Figure 7C). This function may play an oncogenic role for tumor cells residing in hypoxic skin lesions. 

## 3. Discussion

**Figure 8:**
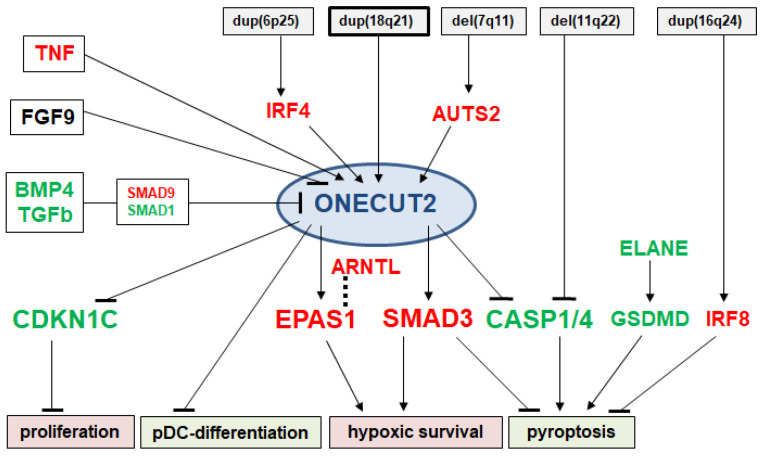
Gene regulatory network around aberrantly expressed *ONECUT2* in BPDCN, summarizing the results of this study. Regulatory relationships are indicated by lines. Upstream regulators including signaling pathways, genomic aberrations, TFs and chromatin factors are shown. Downstream functions include activation of proliferation and hypoxic survival and inhibition of pDC-differentiation and pyroptosis. Gene expression levels in CAL-1 cells are indicated by colors—red: elevated; green: downregulated; black: no alteration.

In this study, we investigated CUT-class homeobox genes in myelopoiesis including DCs. *CUX2* was physiologically expressed in pDCs and silent in cDCs, while *ONECUT2* was aberrantly expressed in BPDCN. Using BPDCN cell line CAL-1 as a model, we characterized the regulatory architecture of *ONECUT2* by identifying its upstream factors and pathways and downstream target genes and functions. These results are summarized in Figure 8, showing a pathogenic gene regulatory network around *ONECUT2* which drives oncogenic activities in this fatal malignancy. 

The founding member of CUT-class homeobox genes was identified in *Drosophila*, where it regulates both cell-type specification and morphogenesis [39,40]. Subsequent analyses of CUT-class homeobox genes in vertebrates and humans confirmed similar functions in several tissues, including the hematopoietic system. For example, CUX1 affects both myeloid and lymphoid differentiation, and CUX2 controls *HOX* gene expression during forelimb development [41,42]. ONECUT1 and ONECUT2 regulate early retinal cell fates, while the latter also regulates intestinal cell differentiation [43,44]. SATB1 supports self-renewal of HSCs inhibiting their differentiation and orchestrates erythroid development via regulation of globin genes [45,46]. Furthermore, its CUT domain mediates interaction with the matrix attachment region, thus organizing intranuclear structures [47]. Collectively, CUT-class homeobox genes are tightly involved in regulation of development and cell differentiation, including immune and blood cells. 

According to their physiological activities, deregulated CUT-class homeobox genes play varied and important roles in cancer [48]. For example, *CUX1* acts as a tumor suppressor in many cancer types, including myeloid malignancies, inhibiting RAS- and PI3K-signalling and activating DNA repair [49,50,51,52]. In contrast, *ONECUT2* acts as an oncogene in prostate and lung cancer [20,21,22]. 

In previous studies, we have proposed several types of TF-code, detailing gene signatures for selected TF groups in the hematopoietic compartment. Examples include the NKL- and TALE-codes, which cover NKL-subclass and TALE-class homeobox gene activities for each progenitor and mature immune cell entity [7,9,10]. In line with these previously published codes, we here describe the myeloid CUT-code, covering CUT-class homeobox gene activities in developing and mature myeloid cell types. Hence, *CUX1* was detected in all entities which may correspond to its reported general impact on myeloid differentiation [42]. *CUX2* expression was restricted to LMPPs and pDCs, indicating that this CUT-class homeobox gene may specifically regulate the differentiation of pDCs and cDCs. *ONECUT2* was physiologically silent in the complete myeloid compartment, demonstrating ectopic activation in BPDCN. In murine hematopoiesis, DCs derive from CDPs or CLPs [13,26,27]. However, the NKL homeobox gene *VENTX* is specifically expressed in human hematopoiesis including cDCs but lacks in the genome of mice and rats [15,53]. Thus, gene activities differ in human and murine hematopoiesis, reminding us not to transfer mouse data to humans without analysis of the corresponding human cell types.

BPDCN model cell line CAL-1 bore the genomic alteration dup(18)(q21-q23) which focally targeted *ONECUT2* at 18q21. This copy number gain likely underlies aberrant *ONECUT2* expression in this cell line. Most BPDCN patients show altered chromosomes, including complex karyotypes and copy number alterations [33]. In addition, CAL-1 harbors the sporadic alteration t(6;8)(p21;q24) which juxtaposes *RUNX2* and *MYC* [34]. In CAL-1, we also detected dup(6)(p21-p25) targeting *IRF4* at 6p25, dup(16)(q22-q24) targeting *IRF8* at 16q24 and a microdeletion at 7q11.22 which eliminates an intragenic component of AUTS2. 

In addition to genomic aberrations, we identified four altered pathways which contributed to *ONECUT2* expression, including BMP4-, FGF9-, TGFb- and TNF-signaling. BMP-signaling inhibits *ONECUT2* in trigeminal sensory neuron differentiation [54], demonstrating that this pathway plays a physiological role in ONECUT2 regulation in some cell types. We failed to detect any regulatory impact for TNFRSF11A (also named RANK); although, this receptor has been shown to activate *ONECUT2* in enterocytes of the small intestine [43]. In T-cells, *ONECUT2* is regulated by TBX21/T-bet [55]. However, this factor is not expressed in CAL-1 (Table S2), probably discounting any similar role in BPDCN. In contrast, we identified elevated IRF4 as a transcriptional activator of *ONECUT2* in CAL-1 where both *IRF4* and *IRF8* are highly expressed and targeted by genomic duplication. These genes play essential roles in development and differentiation of cDCs and pDCs, suggesting aberrant consequences when overexpressed [13,56,57]. 

In breast cancer, ONECUT2 inhibits *CASP3* and activates *BCL2*, which together support survival of these tumor cells [58]. This observation corresponds to our results, demonstrating that ONECUT2 inhibits *CASP1* in BPDCN; although, *ONECUT2* downregulation showed no significant effect on apoptosis. However, pDCs mediate their immune response by production of inhibitory chemokines which contribute to their pyroptotic cell death as observed during inflammation [59]. Regulators and actors in pyroptosis include *CASP1*, *ELANE*, *GSDMD*, *IRF8* and *SMAD3*. These genes showed altered expression levels in CAL-1 (Table S2), collectively indicating suppression of pyroptotic cell death in BPDCN. Therefore, their deregulation may contribute to aberrant survival of BPDCN cells. 

DCs are mobile immune cells present in most tissues. Thus, they are confronted with various environments with a diversity of oxygen levels. Reportedly, hypoxia induces cell death of monocyte-derived DCs [60]. Furthermore, the hypoxic environment reprograms DCs, alters their metabolism and inhibits differentiation and maturation of pDCs, blunting defensive responses to tumor cells [61,62]. These data indicate that normal DCs show substantial responses to hypoxia. Our results indicate that malignant pDCs acquired aberrant activities to escape hypoxia-induced cell death. ONECUT2 activates *SMAD3* and *EPAS1* which mediate survival of tumor cells under hypoxic conditions as shown recently for *SMAD3* in prostate cancer and in this study for *EPAS1* in BPDCN [21]. Furthermore, CAL-1 cells expressed high levels of *ARNTL*, while *ONECUT2*-high BPDCN patients expressed elevated *ARNT*. EPAS1, ARNT and ARNTL are members of the bHLH/PAS-family and are able to interact and cooperate, suggesting functional relevance for their coexpression [38]. Moreover, in endochondral ossification—where cartilage scaffolds are replaced by forming bone—EPAS1 activates expression of *RUNX2* [63]. In BPDCN, the super-enhancer from *RUNX2* is frequently translocated to *MYC*, thus acting as an oncogene-driver in this malignancy [33,34]. Therefore, aberrant EPAS1 activity may plausibly enhance *MYC* expression via the translocated enhancer of *RUNX2* in these cells. 

Finally, we detected elevated *EPAS1* expression in ALK-positive ALCL patients and cell lines. ALCL and BPDCN tumor cells are mostly located in (hypoxic) skin lesions where EPAS1 may support their survival. The availability and application of proven EPAS1-inhibitors, like Belzutifan, for treatment of specific cancer types may promote the usage of these drugs for BPDCN and ALCL as well [64]. A recently formed consortium aims to reveal novel therapies for BPDCN, including CD123/IL3R-directed drug targeting using tagraxofusp [65]. Generally, targeted treatments promise reduced side effects as compared to conventional chemotherapies. However, the pros and cons of novel therapies including Belzutifan have to be validated carefully. Taken together, our data indicate that CUT-class homeobox genes *CUX2* and *ONECUT2* may be useful biomarkers while hypoxia factors EPAS1 and ARNT/ARNTL may serve as novel therapeutic targets for BPDCN (and ALCL). Accordingly, our findings may illuminate diagnosis of BPDCN and uncover actionable therapeutic targets in this intractable malignancy.

## 4. Materials and Methods

### 4.1. Bioinformatic Analyses of Expression Profiling and RNA-seq Data

Expression data for normal cell types were obtained from Gene Expression Omnibus (GEO, www.ncbi.nlm.nih.gov, accessed on 1 November 2023). We analyzed pDC and cDC samples using dataset GSE24759 and the associated online tool GEO2R for their comparison, revealing significant differentially expressed genes [66,67]. Furthermore, we used expression profiling datasets GSE42619, GSE22552, GSE109348, GSE40831 and GSE14879, in addition to RNA-seq dataset GSE69239 and RNA-seq data from The Human Protein Atlas (www.proteinatlas.org, accessed on 1 November 2023) [68]. Gene expression profiling data from BPDCN and AML patients were examined using dataset GSE89565 [69]. For analysis of cell lines, we exploited RNA-seq data from 100 leukemia/lymphoma cell lines (termed LL-100), available at ArrayExpress (www.ebi.ac.uk/arrayexpress) via E-MTAB-7721. Gene expression values are given as DESeq2 normalized count data [70]. ONECUT2 knockdown RNA-seq data are stored at BioStudies (www.ebi.ac.uk/biostudies/) and available via S-BSST1311.

### 4.2. Cell Lines and Treatments

Cell lines are held at the DSMZ (Braunschweig, Germany) and cultivated as described (www.DSMZ.de, accessed on 1 November 2023). All cell lines had been authenticated and tested negative for mycoplasma infection. Modification of gene expression levels was performed using gene specific siRNA oligonucleotides with reference to AllStars negative Control siRNA (siCTR) obtained from Qiagen (Hilden, Germany). SiRNAs (100 pmol) were transfected into 1 × 10^6^ cells by electroporation using the EPI-2500 impulse generator (Fischer, Heidelberg, Germany) at 350 V for 10 ms. Electroporated cells were harvested after 20 h cultivation. Cell treatments were performed using 20 ng/mL recombinant BMP4, TGFbeta, TNF, FGF2 and FGF9 for 20 h and 20 ng/mL GMCSF and IL3 for 40 h (R & D Systems, Wiesbaden, Germany). RNA-sequencing analysis was performed in triplicate of CAL-1 cells treated for 20 h by siRNA-mediated knockdown (Eurofins MWG, Konstanz, Germany) using RNAeasy for RNA extraction (Qiagen) and Bioanalyzer for quality control (Agilent Technologies, Santa Clara, CA, USA). For functional testing, treated cells were analyzed with the IncuCyte S3 Live-Cell Imaging Analysis System (Sartorius, Göttingen, Germany). For detection of apoptotic cells, we additionally used the IncuCyte Caspase-3/7 Green Apoptosis Assay diluted at 1:2000 (Sartorius). The cells were incubated in normoxic (20% oxygen, 5% carbon dioxide) or hypoxic (5% oxygen, 5% carbon dioxide) conditions. Live-cell imaging experiments were performed twice with fourfold parallel tests. 

### 4.3. Polymerase Chain Reaction (PCR) Analyses

Total RNA was extracted from cultivated cell lines using TRIzol reagent (Thermo Fisher Scientific, Darmstadt, Germany). Primary human total RNA derived from HSCs, monocytes and DCs was purchased from Biochain/BioCat (Heidelberg, Germany). cDNA was synthesized using 1 µg RNA, random priming and Superscript II (Thermo Fisher Scientific). Real-time quantitative (RQ)-PCR analysis was performed using the 7500 Real-time System and commercial buffer and primer sets (Thermo Fisher Scientific). For normalization of expression levels, we quantified the transcripts of TATA box binding protein (TBP). Quantitative analyses were performed as biological replicates and measured in triplicate. Standard deviations are presented in the figures as error bars. Statistical significance was assessed by Student’s *t*-test (two-tailed) and the calculated *p*-values indicated by asterisks (* *p* < 0.05, ** *p* < 0.01, *** *p* < 0.001, n.s. not significant). 

### 4.4. Protein Analysis

Western blots were generated by the semi-dry method. Protein lysates from cell lines were prepared using SIGMAFast protease inhibitor cocktail (Sigma-Aldrich, Taufkirchen, Germany). Proteins were transferred onto nitrocellulose membranes (Bio-Rad, München, Germany) and blocked with 5% dry milk powder dissolved in phosphate-buffered saline (PBS). The following antibodies were used: alpha-Tubulin (Sigma, #T6199), CUX2 (Abnova, #H00023316-M03), IRF4 (Origene, #TA351302) and EPAS (MyBioSource, #MBS9127904). For the loading control, blots were reversibly stained with Poinceau (Sigma), and detection of alpha-Tubulin (TUBA) was performed thereafter. Secondary antibodies were linked to peroxidase for detection by Western-Lightning-ECL (Perkin Elmer, Waltham, MA, USA). Documentation was performed using the digital system ChemoStar Imager (INTAS, Göttingen, Germany). Immuno-cytology was performed as follows: Cells were spun onto slides, air-dried and fixed with methanol/acetic acid for 90 s. The ONECUT2 antibody (LSBio, #LS-C499462) was diluted 1:20 in PBS containing 5% BSA and incubated for 30 min. Washing was performed 3 times with PBS. Preparations were incubated with fluorescent secondary antibody (diluted 1:100) for 20 min. After final washing, the cells were mounted in Vectashield (Vector Laboratories, Burlingame, CA), containing DAPI for nuclear staining. Images were captured with an Axion A1 microscope using Axiocam 208 color and software ZEN 3.3 blue edition (Zeiss, Göttingen, Germany). 

### 4.5. Genomic Profiling Analysis

For genomic profiling, genomic DNA of cell lines was prepared by the Qiagen Gentra Puregene Kit (Qiagen). Labelling, hybridization and scanning of Cytoscan HD arrays were performed by the Genome Analytics Facility located at the Helmholtz Centre for Infection Research (Braunschweig, Germany), using the manufacturer’s protocols (Affymetrix, High Wycombe, UK). Data were interpreted using the Chromosome Analysis Suite software version 3.1.0.15 (Affymetrix, High Wycombe, UK) and copy number alterations determined accordingly.

## 5. Conclusions

We detected normal and aberrant activities of CUT-class homeobox genes CUX2 and ONECUT2 in pDCs and BPDCN, respectively. ONECUT2 is located in a pathogenic network which contains novel players in this disease. Thus, our results may contribute to improving diagnostic and/or therapy of this fatal malignancy.

## Figures and Tables

**Table 1:** Target gene analysis for ONECUT2 based on RNA-sequencing data.

Gene	log2-fold	padj
DSCAML1	−3.47	6.10 × 10^−6^
UMPS	−1.52	1.41 × 10^−21^
EPAS1	−1.39	2.22 × 10^−10^
ONECUT2	−1.00	5.89 × 10^−8^
CRACD	−0.85	1.83 × 10^−3^
COG5	−0.61	2.98 × 10^−8^
CACYBP	0.62	1.51 × 10^−3^
GEN1	0.68	2.38 × 10^−2^
BCAS3	1.75	1.65 × 10^−12^
AC069368.1	5.53	5.28 × 10^−7^

## Data Availability

The data presented in this study are openly available as indicated in the text.

## Supplementary Materials

The supporting information can be downloaded at: https://www.mdpi.com/article/10.3390/ijms25052764/s1.
