# Supplementary material for: Transcriptional Landscape of CUT-Class Homeobox Genes in Blastic Plasmacytoid Dendritic Cell Neoplasm
Source: Int J Mol Sci. 2024 Feb 27;25(5):2764. doi: 10.3390/ijms25052764 (PMC10932245; doi:10.3390/ijms25052764)
Supplement: Supplementary file 1 [file ijms-25-02764-s001.zip › Supplementary Figures.pdf]

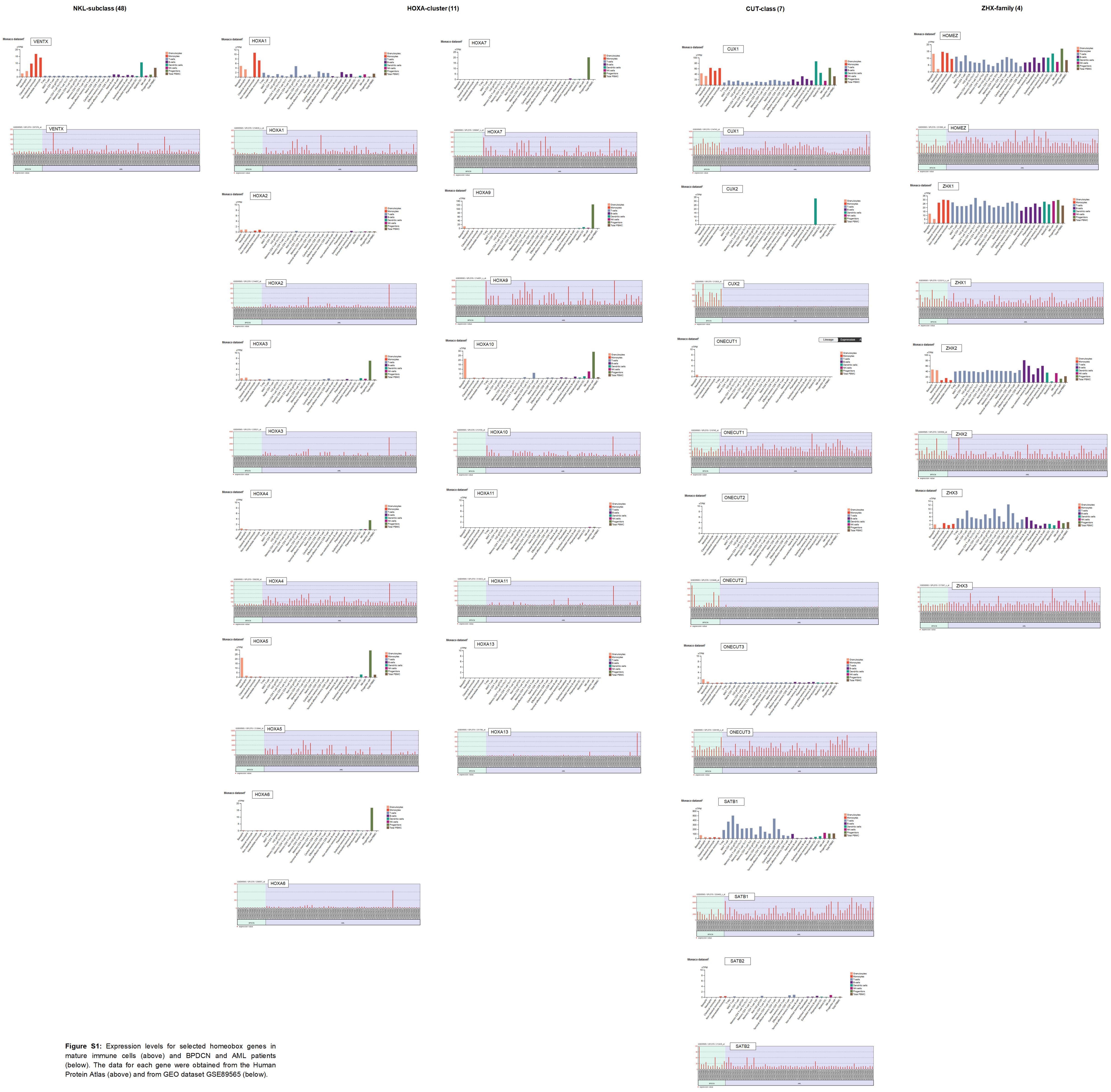

Figure S1: Expression levels for selected homeobox genes in mature immune cells (above) and BPDCN and AML patients (below). The data for each gene were obtained from the Human Protein Atlas (above) and from GEO dataset GSE89565 (below).

HSCs and lymphopoiesis RNA-seq data (GSE69239)

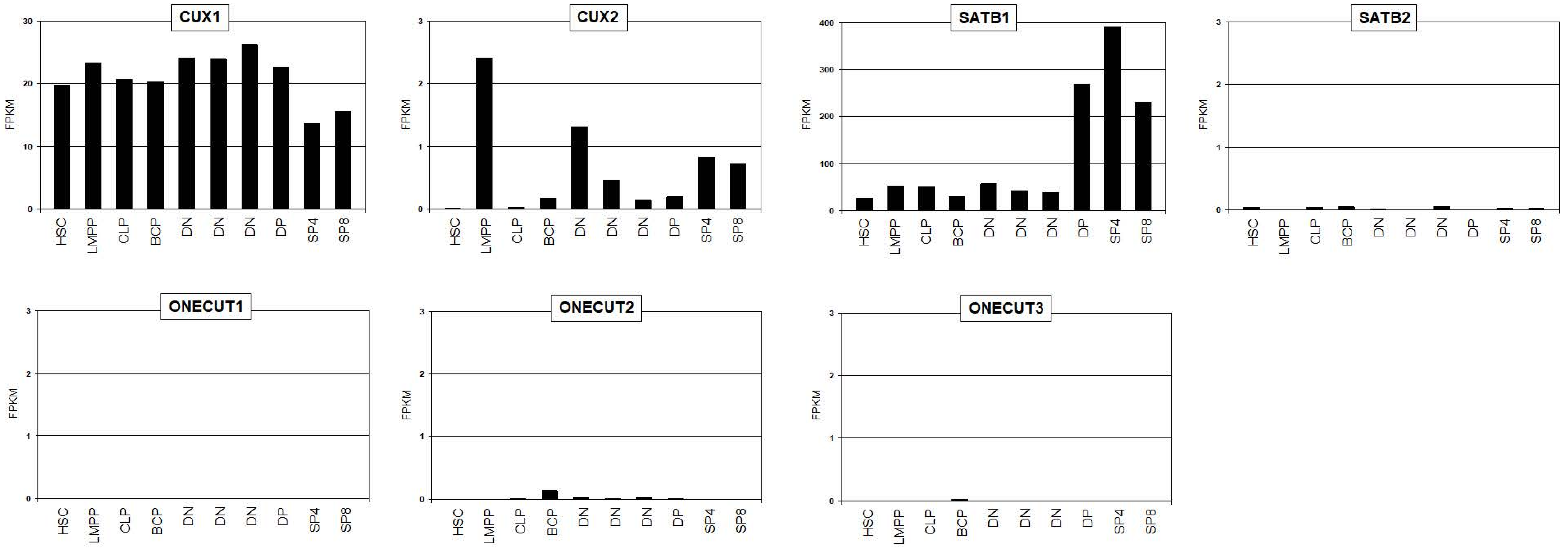

Myelopoiesis GSE42519

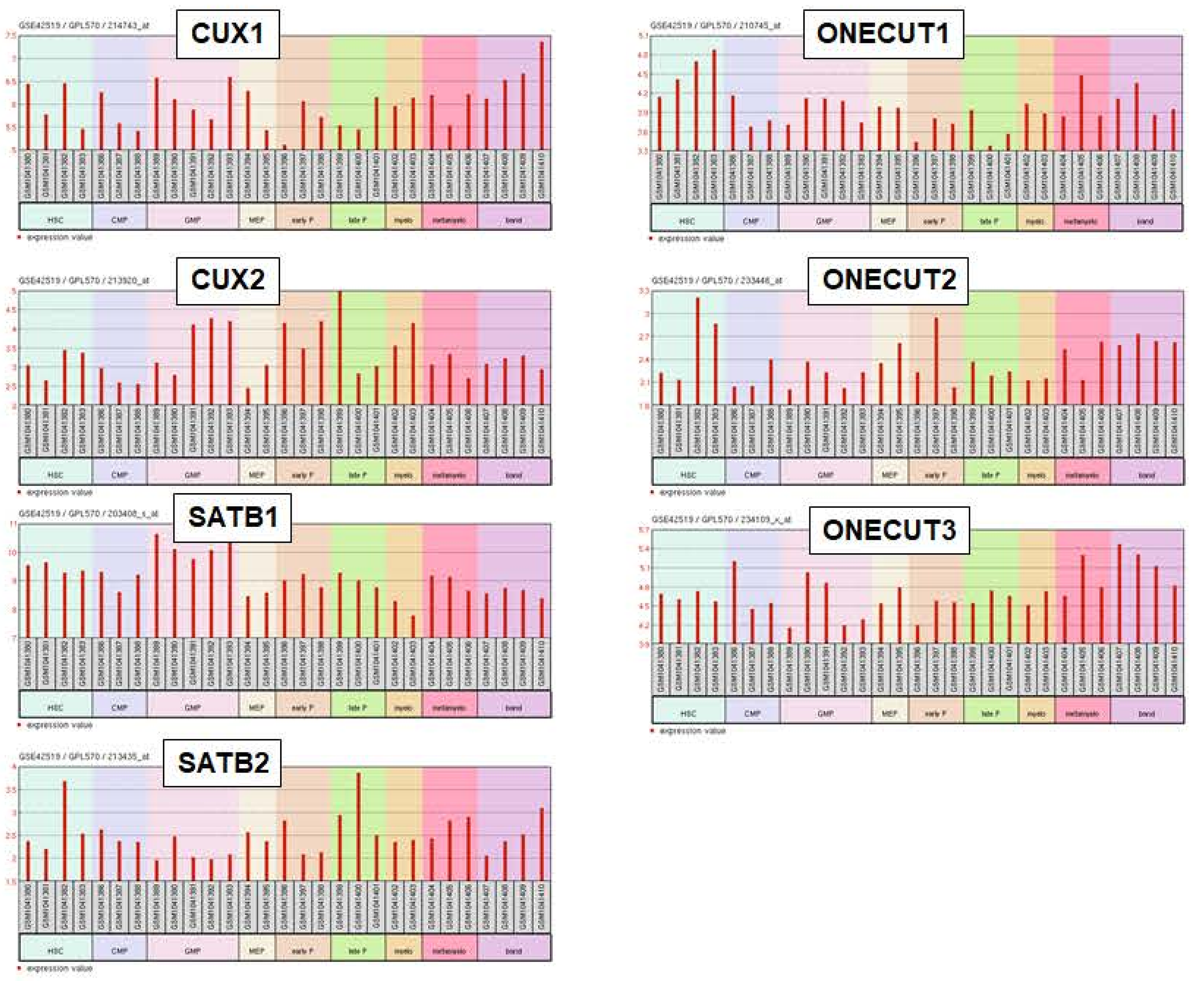

Erythropoiesis GSE22552

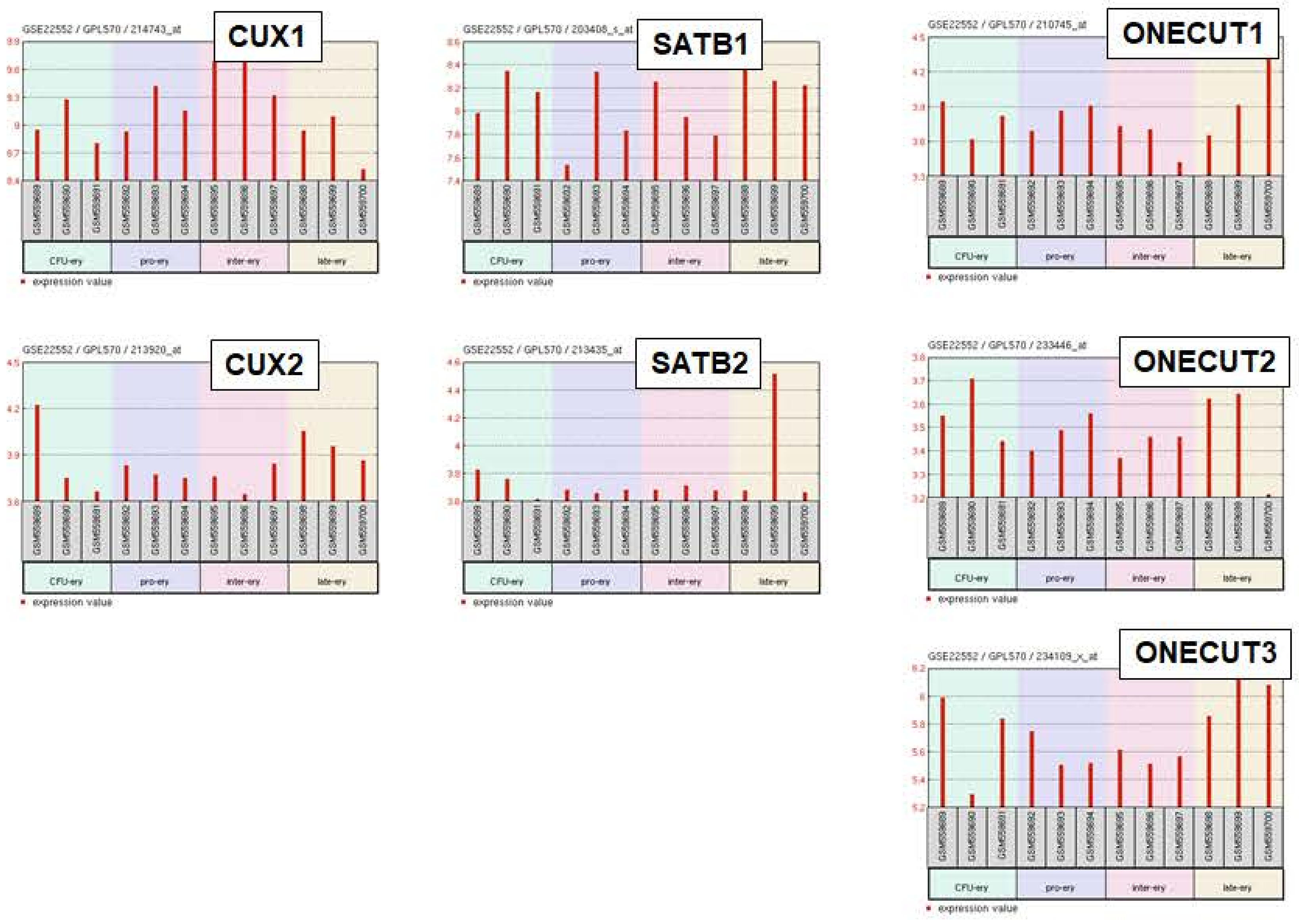

Monocytes, moDC, mast cells, granulocytes GSE109348

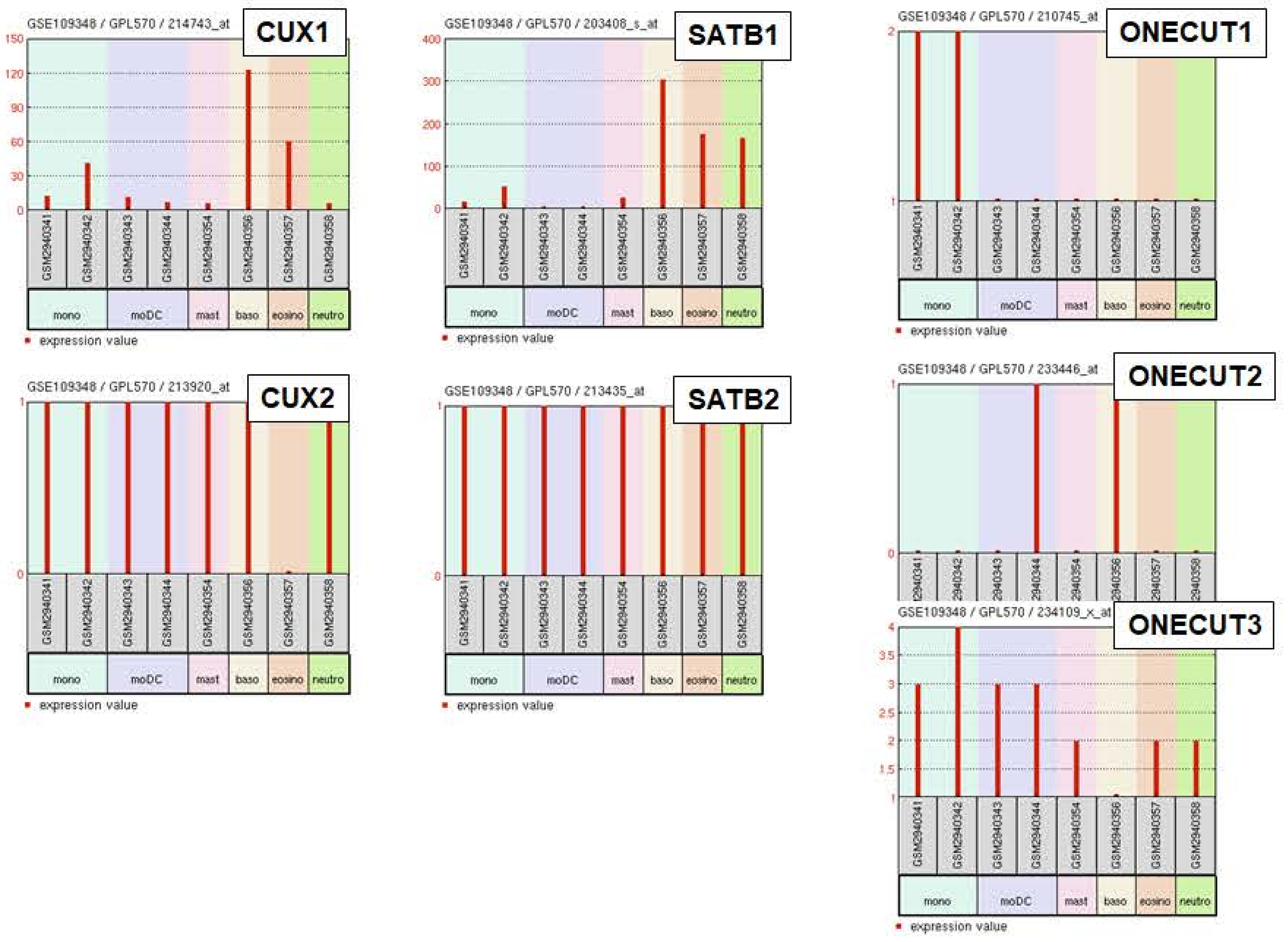

Megakaryocytes GSE40831

Group	Accession	Title	Source name	Cell type
-	GSM1002641	megakaryocytes at day0, biological rep A	umbilical cord blood	CD34-CD41+CD61+CD45- megakaryocytes
-	GSM1002642	megakaryocytes at day0, biological rep B	umbilical cord blood	CD34-CD41+CD61+CD45- megakaryocytes
-	GSM1002643	megakaryocytes at day4, biological rep A	Lin- cell culture derived samples, day4	CD34-CD41+CD61+CD45- megakaryocytes
-	GSM1002644	megakaryocytes at day4, biological rep B	Lin- cell culture derived samples, day4	CD34-CD41+CD61+CD45- megakaryocytes
-	GSM1002645	CFU-M at day0, biological rep A	umbilical cord blood	CD34-CD33+CD13+ colony forming unit-monocytes
-	GSM1002646	CFU-M at day0, biological rep B	umbilical cord blood	CD34-CD33+CD13+ colony forming unit-monocytes
-	GSM1002647	CFU-M at day4, biological rep A	Lin- cell culture derived samples, day4	CD34-CD33+CD13+ colony forming unit-monocytes
-	GSM1002648	CFU-M at day4, biological rep B	Lin- cell culture derived samples, day4	CD34-CD33+CD13+ colony forming unit-monocytes

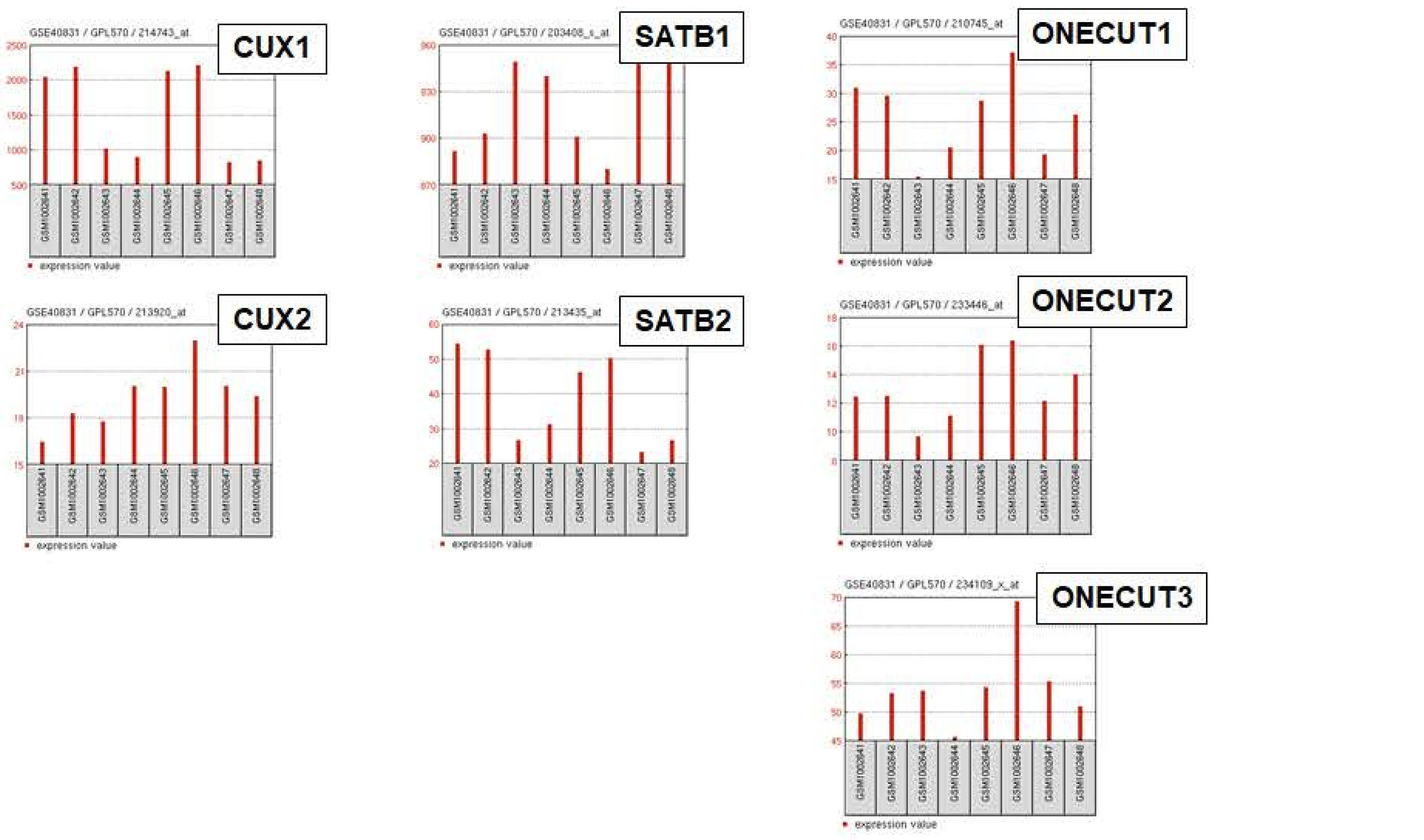

Figure S2: Expression levels of all human CUT-class homeobox genes in early hematopoiesis (dataset GSE69239), myelopoiesis (GSE42519), erythropoiesis (GSE22552), in monocytes, monocyte-derived DCs, mast cells and granulocytes (GSE109348), and megakaryocytes (GSE40831).

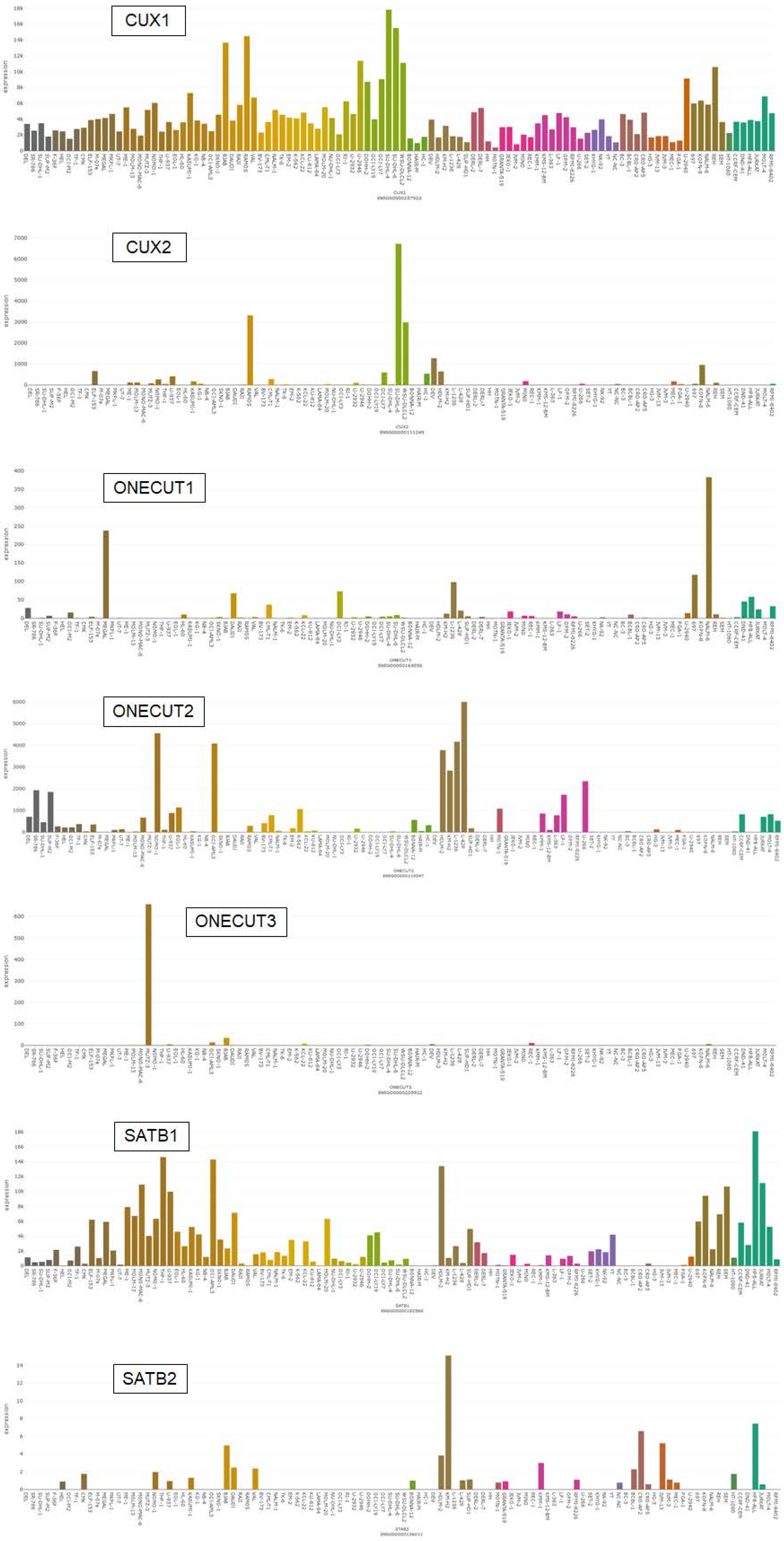

Figure S3: Expression levels of CUT-class homeobox genes in cell lines using RNA-seq dataset LL-100.

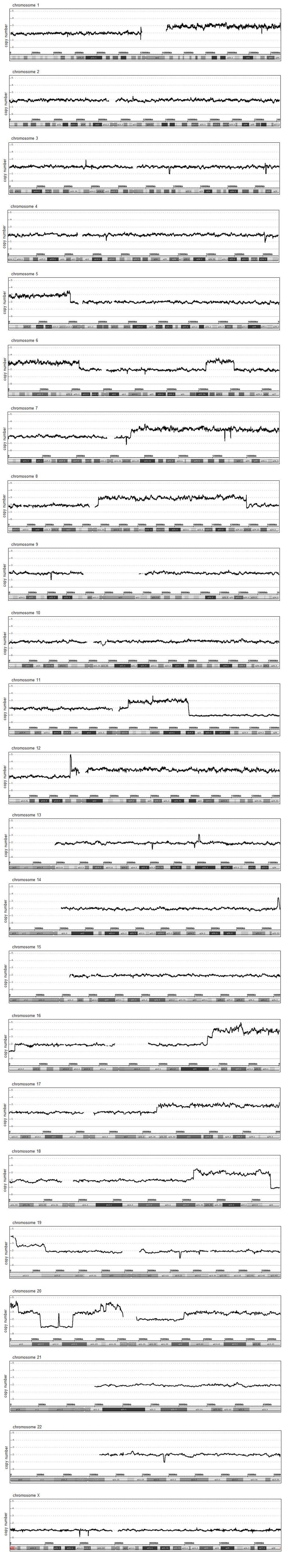

Figure S4: Genomic profiling data for BPDCN cell line CAL-1, showing copy number values for all chromosomes.
